# Synthesis, molecular docking and DFT analysis of novel *bis*-Schiff base derivatives with thiobarbituric acid for α-glucosidase inhibition assessment

**DOI:** 10.1038/s41598-024-54021-z

**Published:** 2024-02-10

**Authors:** Saba Gul, Faheem Jan, Aftab Alam, Abdul Shakoor, Ajmal Khan, Abdullah F. AlAsmari, Fawaz Alasmari, Momin Khan, Li Bo

**Affiliations:** 1 Department of Chemistry, Abdul Wali Khan University, Mardan, 23200 Pakistan; 2 Shenyang National Laboratory for Materials Science, Institute of Metal Research, Chineses Academy of Sciences, Shenyang, 110016 Liaoning China; 3 School of Materials Science and Engineering, University of Science and Technology of China, Shenyang, 110016 Liaoning China; 4 Department of Chemistry, University of Malakand, P.O. Box 18800, Dir Lower, Pakistan; 5 Natural and Medical Sciences Research Center, University of Nizwa, 616 Birkat Al Mauz, PO Box 33, Nizwa, Oman; 6 Department of Pharmacology and Toxicology, College of Pharmacy, King Saud University, 11451 Riyadh, Saudi Arabia; 7 Institute of Catalysis for Energy and Environment, College of Chemistry and Chemical Engineering, Shenyang Normal University, Shenyang, 110034 China

**Keywords:** Thiobarbituric acid, *bis*-Schiff bases, α-Glucosidase inhibition, NMR spectroscopy, Molecular docking, DFT, Biochemistry, Computational biology and bioinformatics, Drug discovery, Chemistry

## Abstract

A library of novel *bis*-Schiff base derivatives based on thiobarbituric acid has been effectively synthesized by multi-step reactions as part of our ongoing pursuit of novel anti-diabetic agents. All these derivatives were subjected to in vitro α-glucosidase inhibitory potential testing after structural confirmation by modern spectroscopic techniques. Among them, compound **8** (IC_50_ = 0.10 ± 0.05 µM), and **9** (IC_50_ = 0.13 ± 0.03 µM) exhibited promising inhibitory activity better than the standard drug acarbose (IC_50_ = 0.27 ± 0.04 µM). Similarly, derivatives (**5**, **6**, **7**, **10** and **4**) showed significant to good inhibitory activity in the range of IC_50_ values from 0.32 ± 0.03 to 0.52 ± 0.02 µM. These derivatives were docked with the target protein to elucidate their binding affinities and key interactions, providing additional insights into their inhibitory mechanisms. The chemical nature of these compounds were reveal by performing the density functional theory (DFT) calculation using hybrid B3LYP functional with 6-311++G(d,p) basis set. The presence of intramolecular H-bonding was explored by DFT-d3 and reduced density gradient (RGD) analysis. Furthermore, various reactivity parameters were explored by performing TD-DFT at CAM-B3LYP/6-311++G(d,p) method.

## Introduction

Due to their various biological applications, Schiff bases, di-imines/*bis*-Schiff bases or azines often created by the condensation of aromatic or aliphatic aldehydes with aniline or aliphatic amines—have become increasingly important in recent decades^1^. Heteroatoms, along with the azomethine or imine connection, are responsible for this unique characteristic^2^. With the general formula R1HC=N–N=CHR2, these compounds feature two double bonds between carbon and nitrogen (–HC=N–)^3^. They are often synthesized by catalytically reacting primary amines with aliphatic/aromatic aldehydes or ketones in an acidic solution. Azine structures can exhibit symmetry (R_1_R_2_ = R_3_R_4_) or asymmetry (R_1_R_2_ ≠ R_3_R_4_), contingent upon the type of substituent present^4^. Di-imines, among others, have garnered attention due to their diverse properties, including anti-inflammatory^5^, anti-tumor^6^, antioxidant^7^, anti-bacterial^8^, anti-parasitic^9^, and anti-glycation^10^ effects.

**Figure 1:**
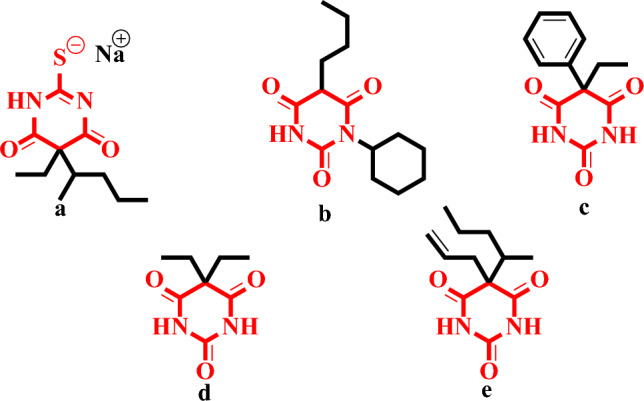
Commercially available drugs (**a**–**e**) containing barbituric acid nucleus.

This structural motif is not unique to this class of compounds alone. Barbiturates, another class of compounds with significant pharmacological importance, also possess a heterocyclic structure derived from barbituric acid. Barbiturates have played a key role in the history of pharmacology, particularly as sedatives and hypnotics^11,12^. The sulfur-containing analogue of barbituric acid, thiobarbituric acid (TBA), exhibits unique structural features and diverse pharmacological activities, including anti-oxidant^13^, anti-cancer^14^, anti-glycation^15^, anti-convulsant^16^, anti-tumor^17,18^, and anti-hypnotic^19^. Barbituric acid derivatives, including sodium pentothal (a), bucolome (b), phenobarbital (c), veronal (d), and seconal (e) (Fig. 1) are the most popular drug used in the market^20^. For medicinal chemists, barbitals and phenobarbitals became attractive targets due to their intriguing mechanisms of action against epilepsy. They have also been studied as potential as Parkinson^21^, anti-cancer and anti-AIDS agents^22,23^. Frequently TBA is utilized as a synthon in the development of anti-cancer medicines due to its ability to generate hydrogen bonding connections with therapeutic targets, which is a result of the significant biological activities of these analogues^24,25^.

This structural knowledge becomes particularly relevant in the context of diabetes mellitus (DM), a chronic endocrine disease that significantly influences the regulation of glucose, electrolytes, fats, proteins, and water metabolism^26,27^. This disorder fits to a class of metabolic disorders described by hyperglycemia, reducing from inadequate insulin production by the pancreas or insufficient cellular receptiveness to insulin^28,29^. The primary therapeutic objective for managing diabetes is the reduction of postprandial hyperglycemia. Particularly, α-glucosidase is the key enzyme responsible for catalyzing the conversion of starch, digestion of carbohydrates and disaccharides into glucose^30^. This enzyme is crucial in the development of drug to treat diabetes, function as competitive antagonists against α-glucosidase enzyme, integral to carbohydrate digestion if the small intestine^31,32^. Inhibiting this enzyme contributes to the deceleration of carbohydrate digestion, highlighting the significance of such targeted approaches in diabetes care^33,34^. The prevalence of diabetes mellitus has reached alarming levels globally, necessitating continuous efforts to discover innovative therapeutic agents^35^.

The promising avenue is the development of compounds targeting α-glucosidase, a key enzyme involved in the digestion of carbohydrates. Derivatives of thiobarbituric acid have revealed miscellaneous pharmacological activities^36,37^, prompting the exploration of their potential as α-glucosidase inhibitors. However, the synthesis of novel *bis*-Schiff base derivatives bearing thiobarbituric acid nucleus remains underexplored. Our current study aims to address this gap by synthesizing a library of novel *bis*-Schiff base derivatives and to evaluate their in vitro α-glucosidase inhibitory potential^38^. The rationale behind this endeavor lies in the potential of these analogues to offer enhances inhibitory effects, contributing valuable insights to the development of novel therapeutic agents for diabetic management^39^. Through a comprehensive screening process, we seek to identify potential lead candidates with promising α-glucosidase inhibitory properties, laying the foundation for further investigations and drug development in the field of diabetes therapeutics^40^.

## Results

### Chemistry

**Figure 2:**
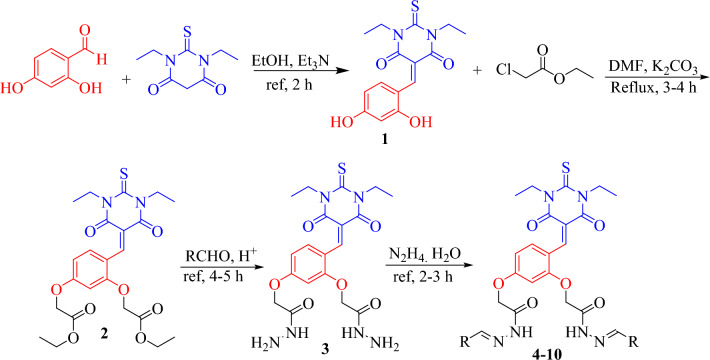
Synthesis of *bis*-Schiff base derivatives based on 1,3-diethyl-2-thiobarbituric acid (**4**–**10**).

In continuous efforts to discover anti-diabetic agents our group has successfully synthesized a library of novel *bis*-Schiff base derivatives based on thiobarbituric acid through multi step reactions. In the first step, 2,4-dihydroxybenzaldehyde was refluxed with 1,3-diethyl-2-thiobarbituric acid in ethanol solvent containing catalytic amount of triethylamine for 2 h with constant stirring to get compound **1**. In the second step, compound **1** was further refluxed with ethyl chloroacetate in DMF solvent having potassium carbonate for 3–4 h to obtain compound **2**, which was further treated with excess of hydrazine hydrate in ethanol solvent to get the *bis*-hydrazide **3** in better yield. Finally, a number of aromatic aldehydes were refluxed with compound **3** for 4–5 h in absolute ethanol having catalytic amount of acetic acid to obtain *bis*-Schiff bases (**4**–**10**) (Fig. 2). Thin layer chromatographic technique was used to know the formation of products in solvent system of *n*-hexane and ethyl acetate (7:3). After the reactions completions, these mixtures were decanted to a beaker having cold distilled water. The appeared colored precipitates were filtered, washed with hot water followed by *n*-hexane, recrystallized with absolute ethanol to get the products in pure form. All the product compounds were structurally deduced through HR-ESI-MS, ^13^C-, and ^1^H-NMR spectroscopy.

The ^1^H-NMR spectra of the synthesized compounds (**4**–**10**) revealed signals for several methine (–CH), methylene (–CH_2_), methyl (–CH_3_), and NH protons. In the down field region of the spectrum, singlet signals of two proton integrations appeared at 12.88–11.79 were due to the –NH protons. Similarly, another singlet signals resonated in the range 9.93–7.98 was assigned to the methine (=CH) protons. In the spectrum, signals of the aromatic protons were seen at 8.39–6.98 and 5.54–5.30 were due to the methylene protons of barbituric acid, while signals observed at 3.78–3.75 were assigned to the methyl protons. The ^13^C-NMR (DEPT and Broad Band) spectra of these derivatives exhibited signals for methyl, methylene, methine, and quaternary carbon atoms. The signal observed between 170.5 and 169.9 in the down field portion of the spectrum was caused by the carbonyl carbon. The signals at 165.2–159.9 were created in the same way by a quaternary carbon containing oxygen and nitrogen atoms. The signal of the methylene group, on the other hand, resonates at 66.6–66.3. Furthermore, molar masses of these analogues were confirmed with the help of HR-ESI-MS spectra showing the molecular ion peaks.

### In-vitro α-glucosidase inhibitory activity

All the synthesized derivatives (**4**–**10**) were assessed for their in vitro a-glucosidase inhibitory potential and compared with the reference drug acarbose (IC_50_ = 0.27 ± 0.04 µM). In this study, all the derivatives exhibited excellent to significant inhibition in the range of IC_50_ values of 0.10 ± 0.05 to 0.52 ± 0.02 µM, amongst them, compound **8** (IC_50_ = 0.10 ± 0.05 µM), and **9** (IC_50_ = 0.13 ± 0.03 µM) attributed the most promising inhibitory activity many times better than the standard acarbose. Similarly, compounds **5**, **6**, **7**, **10** and **4** displayed significant to good activity with IC_50_ values of 0.32 ± 0.03, 0.36 ± 0.04, 0.39 ± 0.08, 0.42 ± 0.07 and 0.52 ± 0.02 µM respectively (Table 1).

**Table 1:** α-Glucosidase inhibitory activity of the synthesized compounds (**4**–**10**).


Comp no.	R	IC_50_ ± SEM (µM)	Comp No	R	IC_50_ ± SEM (µM)
**4**		0.52 ± 0.02	**8**		0.10 ± 0.05
**5**		0.32 ± 0.03	**9**		0.13 ± 0.03
**6**		0.36 ± 0.04	**10**		0.42 ± 0.07
**7**		0.39 ± 0.08	Acarbose		0.27 ± 0.04

## Discussions

### Structure activity relationship study

**Figure 3:**
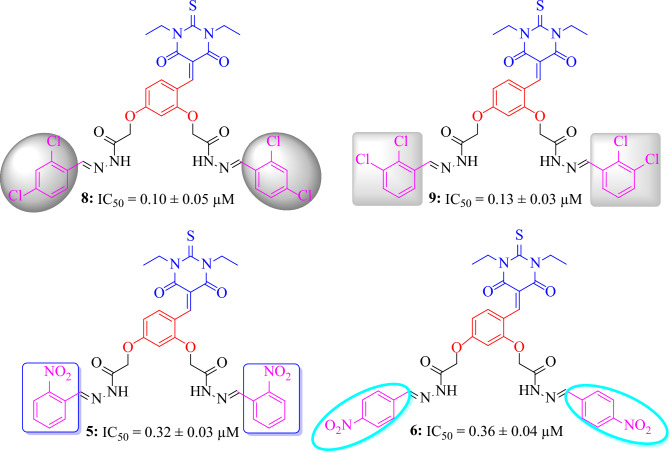
Most active inhibitors among the series.

It's important to note that all compounds were promising α-glucosidase inhibitors, demonstrating that each structural component contributes to the inhibitory activity. Since the aceto-hydrazide moiety, pyrimidine, and benzene ring are identical in all of the compounds, the limited structure–activity relationship (SAR) study is justified by the presence of distinct characteristics, such as various patterns of aryl ring substitution. In the synthesized derivatives, compound **8** (IC_50_ = 0.10 ± 0.05 µM) was the most potent inhibitor of α-glucosidase enzyme. The potency of this compound could be due to the presence of electron withdrawing chlorine atoms attached to the benzene ring at *ortho* and *para* position. A very slight decline occurs in the activity of compound **9** (IC_50_ = 0.13 ± 0.03 µM) could be due to the change of one chlorine atom from para to *meta* position of the benzene ring. Similar to this, the less activity of compound **10** (IC_50_ = 0.42 ± 0.07 µM) might be due to the removal of one chlorine atom from the *ortho* position of the benzene ring from compound **9** (Fig. 3). By comparing compounds **5** (IC_50_ = 0.32 ± 0.03 µM) with **6** (IC_50_ = 0.36 ± 0.04 µM) and **7** (IC_50_ = 0.39 ± 0.08 µM), the highest activity of compound **5** could be due to the attachment of nitro group at *ortho* position. Furthermore, change of position from *ortho* to *para* and *meta* position of the nitro group may be responsible in decreasing the activities of compounds **6** and **7** respectively. However, compound **4** (IC_50_ = 0.52 ± 0.02 µM) showed less inhibitory activity among the series due to the attachment of electron donating hydroxyl group at *para* position of the benzene ring. Our structure activity relationship study showed that compounds having electron-withdrawing substituents displayed superior inhibitory activities. This correlation underscores the significance of these substituents in enhancing the potency of the product compounds. Moving forward, these insights provide a valuable foundation for optimizing future drug candidates in this compounds library.

### Geometries of the compounds

**Figure 4:**
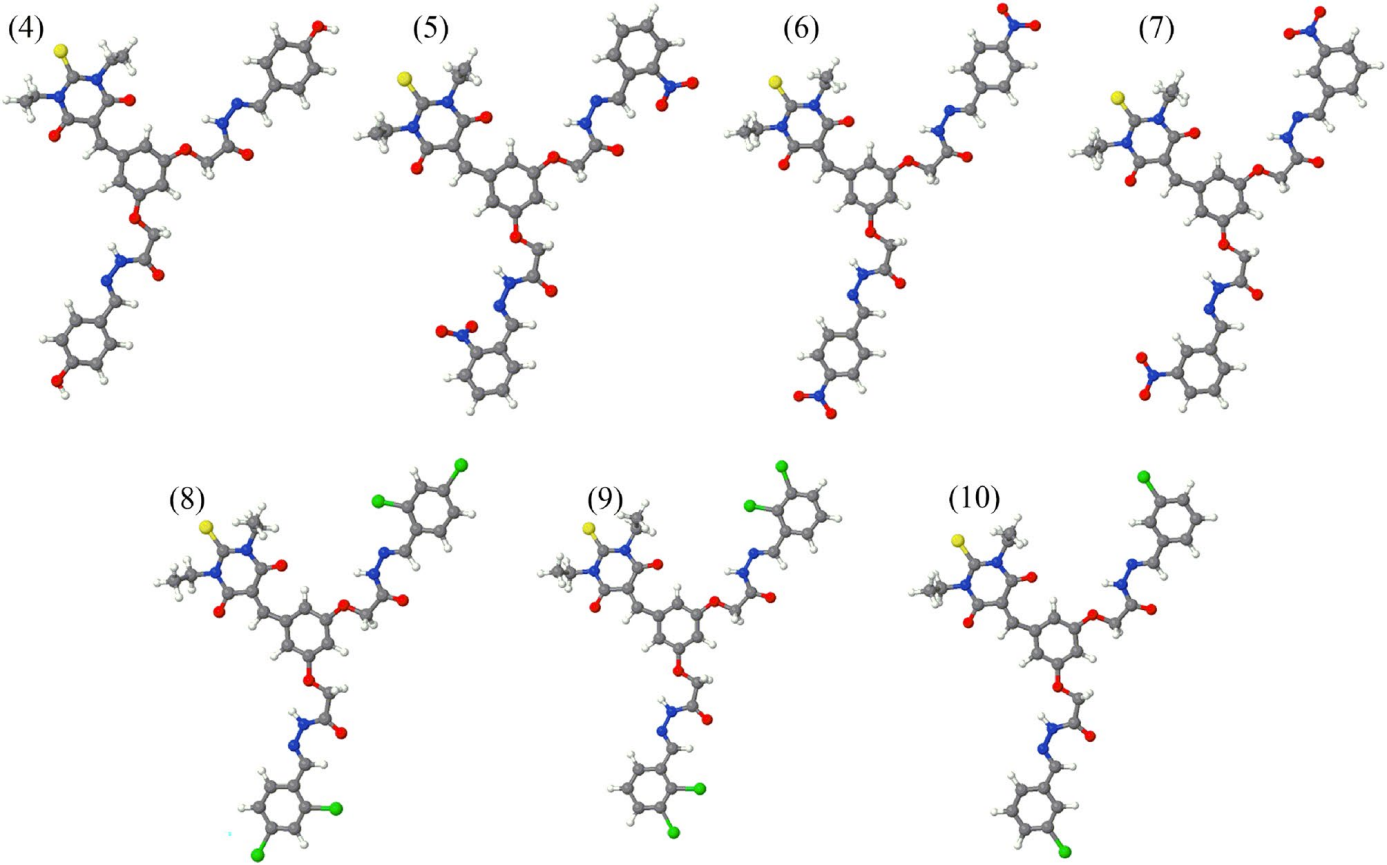
Optimized geometries of synthesized compounds.

After the synthesis, all the compounds were fully optimized to ensure the structural stability. The optimizations were performed at B3LPY/6-311++G(d,p) of DFT method. The optimized structures are shown in Fig. 4.

### NMR analysis

The NMR calculations were performed both experimentally and theoretically. The theoretical chemical shift values were simulated downfield from TMS at B3LYP/6-31+G(2d,p) method. The ^1^H-NMR spectrum reveals distinctive chemical shifts for various functional groups. Notably, in the –CH= group, the experimental and calculated values are reasonably congruent, with a subtle variation observed. This suggests that the theoretical calculations effectively capture the electronic environment of functional groups. The most downfield chemical shift value was found for this group from 11.89 to 9.90 ppm. In contrast, the simulated ^1^H-NMR peak for this group was found the downfield from 10.9 to 9.90 ppm range. The DFT and RGD analysis reveals the presence of intramolecular hydrogen bonding which makes the chemical shift downfield. Similarly, the –CH=N– group exhibit the ^1^H-NMR peak from 10.9 to 9.90 ppm range while the calculated chemical shifts values were found downfield from 9.35 to 9.30 ppm. The –CH_2_ group also display noteworthy agreement between the experimental and theoretical values. The experimental value for –CH_2_ was found from 5.10 to 4.28 ppm downfield from TMS, whereas, the calculated values observed from 4.84 to 4.60 ppm. The chemical environment of aromatic protons is different due to the presence of heteroatoms within the ring. Due to the presence of heteroatoms ^1^H-NMR chemical shift values were observed form 8.31–6.14 ppm experimentally, while the calculated values were found from 8.8 to 6.33 ppm. The detailed ^1^H-NMR chemical shift values are listed in Table 2.

**Table 2:** Calculated and experimental NMR chemical shift values.

Group	Exp	Comp-4	Comp-5	Comp-6	Comp-7	Comp-8	Comp-9	Comp-10
		Calc	Calc	Calc	Calc	Calc	Calc	Calc
^1^H-NMR								
–CH=	11.89–9.90	10.9	9.90	10.66	10.71	10.6	10.68	10.66
–CH=N–	9.35–9.30	10.8, 10.5	11.0, 10.5	10.8, 10.71	10.78, 10.73	10.9, 10.80	11.0, 10.84	10.86, 10.75
–NH	10.08–8.94	9.6, 9.22	9.9, 9.5	10.09, 9.65	10.0, 9.6	10.0, 9.5	10.0, 9.56	9.94, 9.44
–CH_2_	5.10–4.28	4.71–4.59	4.77–4.6	4.78–4.6	4.84–4.62	4.7–4.63	4.76–4.63	4.75–4.63
Ar–H	8.31–6.14	8.40–6.36	8.8–6.4	8.8–6.32	8.9–6.33	8.5–6.36	8.57–6.36	8.58–6.37
^13^C-NMR	168.3–39.0	190.0–48.0	190.5–48.30	190.7–49.50	190.5–49.11	190.5–49.4	190.5–49.6	190.6–48.9

Turning to the ^13^C-NMR spectrum, the experimental values for carbon atoms within the 168.3–39.0 ppm range. However, the calculated chemical shift values were observed within 190.0–49.60 ppm. In summary, the comparison between experimental and calculated chemical shift values across different functional groups in both ^1^H- and ^13^C-NMR spectra suggests that the DFT calculations provide a reliable approximation of the molecular structure and electronic characteristics of the studied compounds.

### RGD analysis

**Figure 5:**
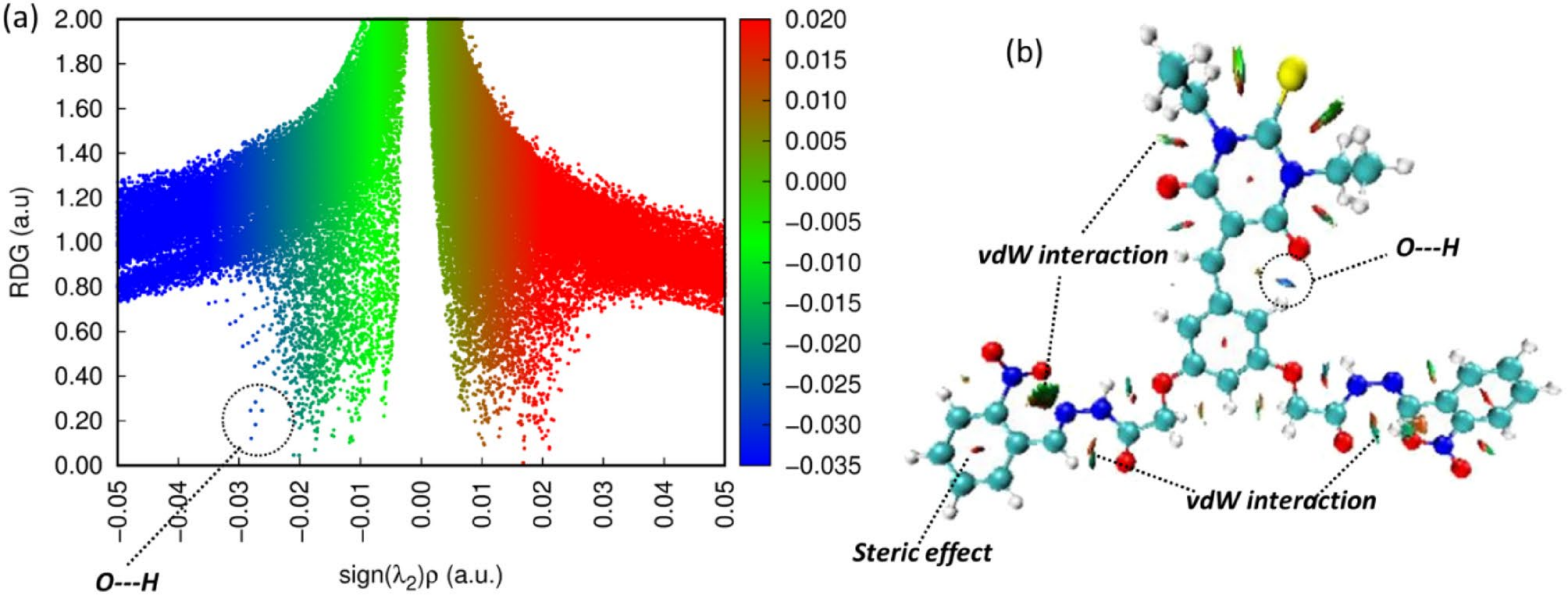
(**a**) Indicate the 2D RGD scatter plot while (**b**) indicate the 3D isosurfaces.

To know about the intermolecular/intramolecular interaction, the RGD analysis is a useful technique^41^. The RGD scatter plot provides the presence of different types of interactions i.e., hydrogen bonding, van der Waals (vdW) and steric effect in the synthesized compounds as shown in Fig. 5a. The scatter plot and 3D isosurfaces indicates the nature of non-covalent interactions associated with the values of sign (λ_2_)ρ values.

On the 2D scatter plot the sign (λ_2_)ρ value is associated with different types of interactions. The deep blue color on the scatter plot signifies a noticeably negative value of (λ_2_)ρ, indicating the presence of attractive forces such as hydrogen bonding or dipole–dipole interactions. In contrast, when observing the scatter graph, a positive (λ_2_)ρ value shows as a red color, pointing to the presence of repulsive forces. Conversely, a value close to zero but remaining negative is as indicated by a green color, indicating the presence of van der Waals interactions. On the scatter plot an observed positive peak range from 0.01 to 0.05 au in the red region signifies the presence of repulsive interaction which is due to steric effect. Furthermore, the range from − 0.01 to 0.01 au on the scatter plot indicates the presence of vdW interactions. The most important peak found in the RGD analysis is the peak that’s range from − 0.02 to − 0.05 au. This peak signifies the presence of strong attractive interaction such hydrogen bonding as found in the synthesized compounds. Figure 5 provides the valuable insights into the nature of attractive, repulsive forces, and specific interactions such as hydrogen bonding and van der Waals forces within the synthesized compounds.

### Frontier molecular orbital (FMO) analysis

For the FMO analysis, TD-DFT calculations were performed using the CAM-B3LYP functional with a 6-311++G(d,p) basis set. The FMO is useful technique used to know about the chemical nature of newly synthesized compounds. In FMO analysis, the HOMO and LUMO energies play a crucial role. The energy gap between these two states (ΔE_gap_) was calculated as follows:

1 $${\Delta {\text{E}}}_{{\text{gap}}}={{\text{E}}}_{{\text{LUMO}}}-{{\text{E}}}_{{\text{HOMO}}}$$

**Figure 6:**
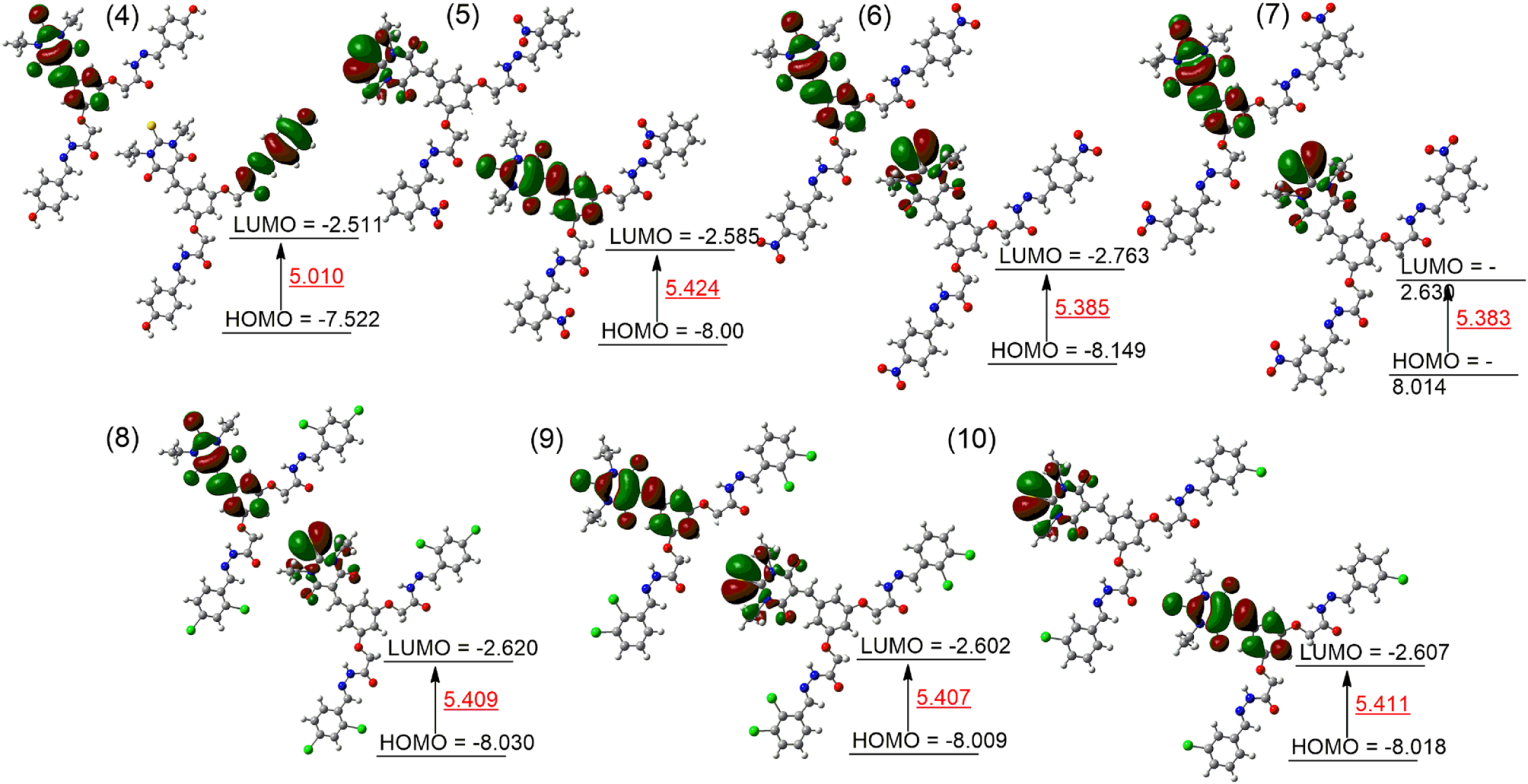
The HOMO and LUMO energies with energy gap between the two states are presented in red color in (eV).

These energy levels provide valuable information about electron distribution, leading to the determination of various properties as listed in Table 3. The energy difference (ΔE_gap_) between HOMO and LUMO is a key factor for understanding a compound's chemical behavior. Compounds with larger energy gaps are less reactive and more stable because they need more energy for electronic excitation. On the other hand, compounds with smaller energy gaps are more reactive, requiring less excitation energy. In this study, the ΔE_gap_ for the synthesized compounds ranged from 5.010 to 5.424 eV. The lowest energy was found for compound 4 as 5.010 eV which indicate a slight higher reactivity. In contrast, the compound 5 shows the highest energy gap of 5.424 eV which shows less reactivity as compared to the compounds. Additionally, compounds 8 and 9 exhibited similar energy differences at 5.409 and 5.407 eV, as shown in Fig. 6.

**Table 3:** Chemical reactivity parameters obtained from TD-DFT analysis.

Parameters	Comp-4	Comp-5	Comp-6	Comp-7	Comp-8	Comp-9	Comp-10
E_HOMO_	− 0.276	− 0.294	− 0.299	− 0.294	− 0.295	− 0.294	− 0.294
E_LUMO_	− 0.092	− 0.095	− 0.101	− 0.096	− 0.096	− 0.095	− 0.095
ΔE	0.184	0.199	0.197	0.197	0.198	0.198	0.198
IP	0.276	0.294	0.299	0.294	0.295	0.294	0.294
EA	0.092	0.0950	0.101	0.096	0.096	0.095	0.095
x	0.184	0.194	0.200	0.195	0.196	0.195	0.195
η	0.0920	0.0996	0.0989	0.0989	0.0994	0.0993	0.0994
σ	10.861	10.033	10.105	10.109	10.060	10.064	10.057

In the FMO calculations, several reactivity parameters were determined such as, ionization energy (IP), electron affinity (EA), electronegativity (x), chemical hardness (η) and softness (σ). The ionization energy gives the information about the removal of an electron from a compound and is linked to E_HOMO_. Ionization energy for the synthesized compounds ranged from 0.276 to 0.299. The compound **4** shows the lowest ionization energy while the compound **6** shows the highest ionization energy of 0.299. The lowest ionization energy of compound **4** suggests easier electron removal compared to other compounds.

The electron affinity (EA) is another important parameter in the chemical reactivity. The electron affinity is the reverse of ionization energy and is define as the amount of energy released by addition of an electron to compound. In this work, the calculated electron affinity values were noted from 0.092 to 0.101. The compound **4** indicated low amount of energy released by the addition of an electron, while reverse is observed for compound 6, which indicate the highest amount of energy release at 0.101.

Additionally, the molecular polarizability can be determined through softness and hardness. A molecule exhibits increased polarizability when it possesses a higher softness value along with a lower hardness value. Among the synthesized compounds, compound **4** is more polarizable due to its low hardness and high softness values. Detailed FMO parameters are listed in Table 3. Overall, the FMO analysis not only explained the electronic structure and reactivity of the synthesized compounds but also provided a comprehensive understanding of their chemical nature.

### Molecular electrostatic potential (MEP) analysis

**Figure 7:**
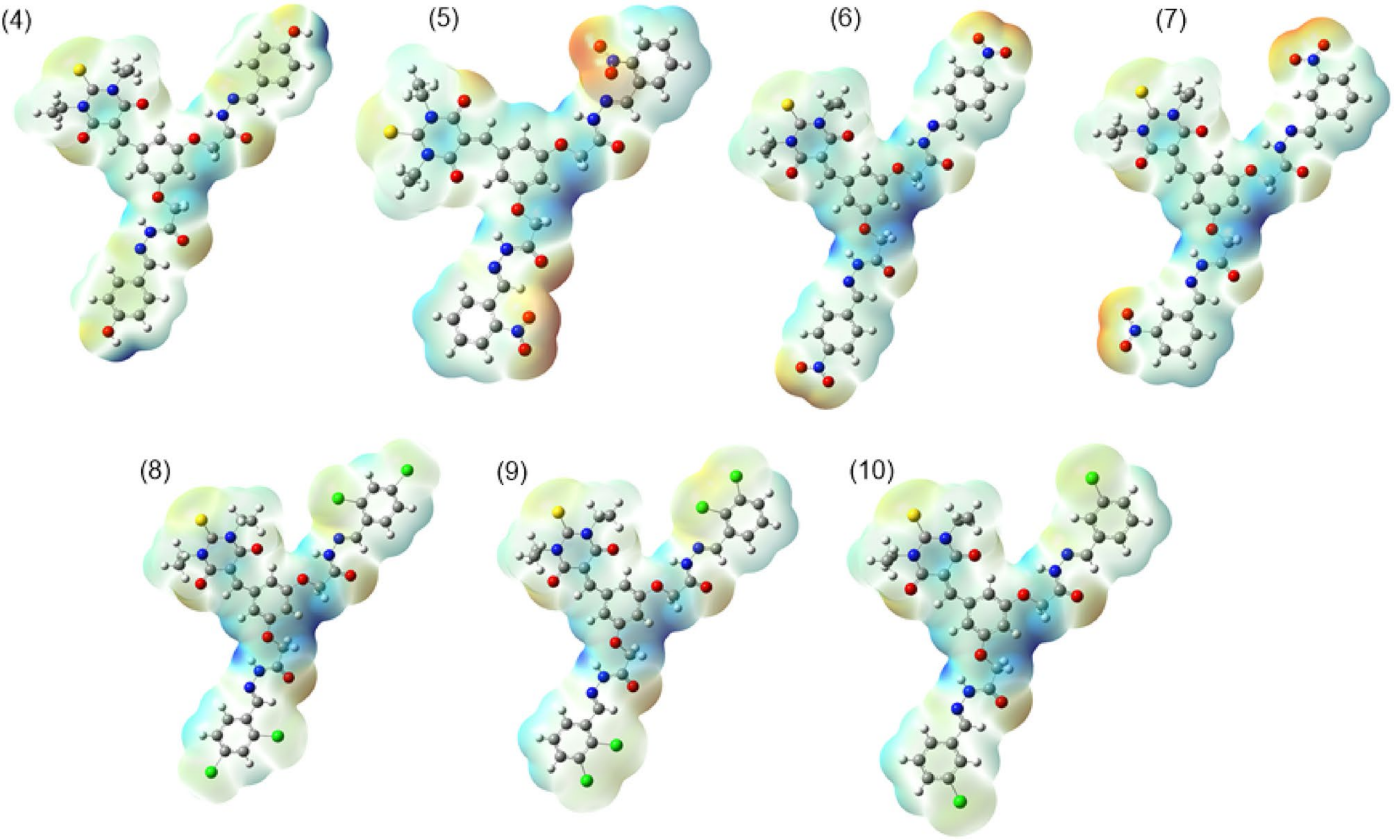
The MEP representations of synthesized compounds.

To identify the electrophilic and nucleophilic sites of synthesized compounds, the MEP analysis was performed at TD-DFT method. The MEP analysis is associated with charged particles within a compound. The blue color in the MEP Fig. 7 indicate the electron deficient region and is considered is a favorable position for nucleophilic attack. Conversely, the regions in red indicate higher electron density, signifying a higher concentration of electrons. These areas are regarded as the preferred sites for electrophilic attacks.

### Molecular docking

The molecular docking of synthesized compounds with α-glucosidase was performed by using the Auto Dock Vina package. The docking study helps to find the binding modes of synthesized compounds (ligands) with amino acids of protein. The synthesized compounds show different interaction with α-glucosidase. The protein structure of α-glucosidase was taken from Protein Data Bank (PDB) at (https://www.rcsb.org/structure/3WY1) with PDB Entry-3WY1.

**Figure 8:**
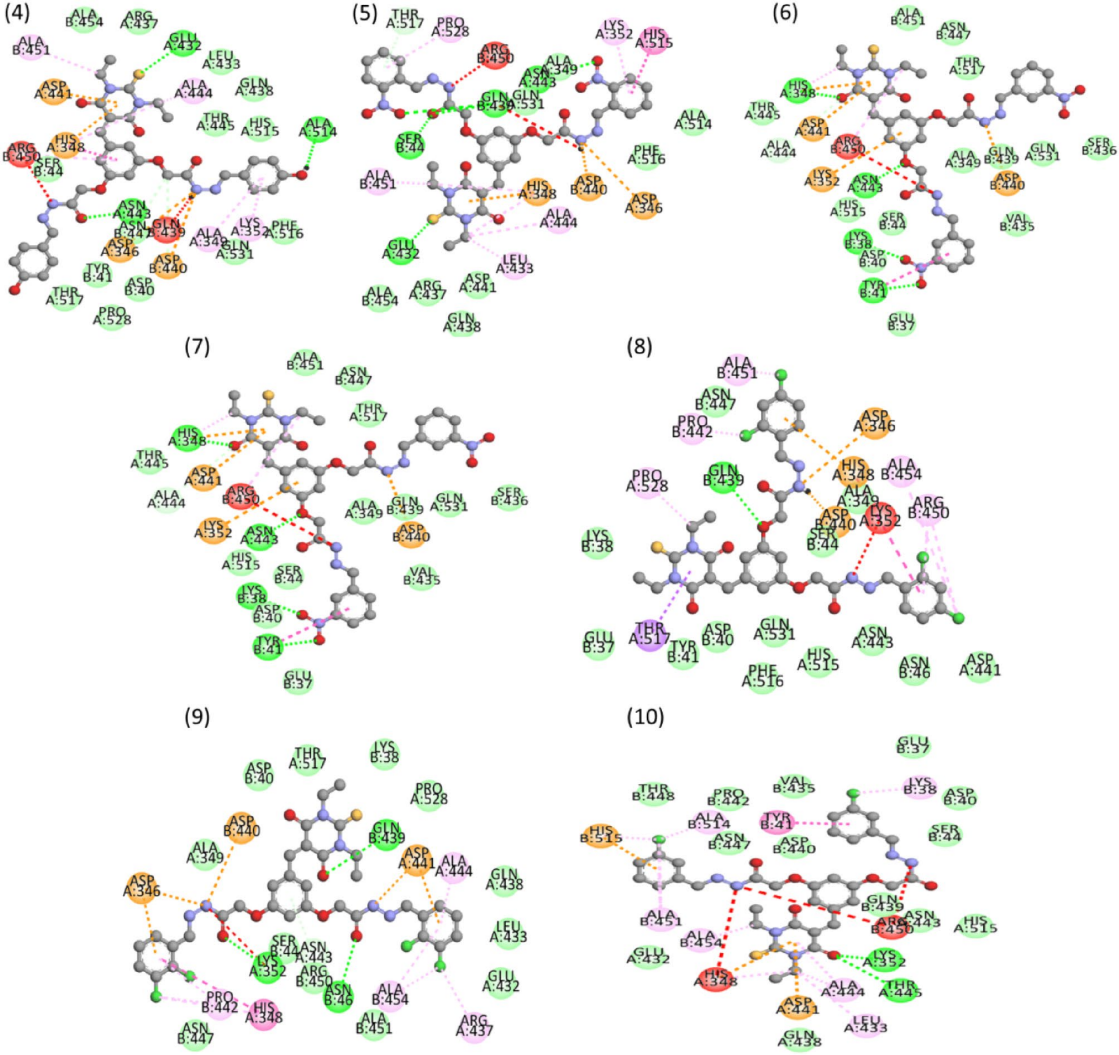
The interaction between amino acids of α-glucosidase with targeted compounds.

In simulation, the water molecules were excluded, and any absent hydrogen atoms in the protein were added using Autodock Tools. The PDB structures of the synthesized compounds (ligands) were formed in pdb format based on the Gaussian optimized structures. The cubic grid box was made with dimensions of 3.391, 1.310, and − 8.362 Å in the x, y, and z-directions respectively. The grid box was centered on the middle of targeted compounds under docking. Grid maps were generated using the AutoGrid Tools with a grid spacing of 0.375 Å. The number of grid points in each direction (x, y, and z) was taken as 40. Additionally, PyMOL 2.5.4 was employed to visualize the interactions between the protein and ligands, while Biovia Discovery Studio was utilized for the 2D representation of protein–ligand interactions, as shown in Fig. 8. The simulation reveals different types of interactions between amino acid and ligands, such as conventional H-bonding, van der Waals, unfavorable positive-positive, attractive and π-interactions as shown in Table 4.

**Table 4:** Representation of protein–ligand interactions.

Compds	Binding affinity (kcal/mol)	Types of interactions				
		Conventional H-bonds	Van der Waals	Unfavorable positive-positive	Attractive charges	π*-interactions
4	− 10.1	GLU.432, ALA.514, ASN.443	ALA.454, ARG.437, LEU.433, GLN.438, THR.445, HIS.515, SER.44, TYR.41, ASP.40, THR.517, PRO.528, GLN.531, PHE.517, PRO.528, GLN.531, PHE.516	ARG.450, GLN.439	–	ALA.451, ALA.444, ALA.349, LYS.352, ASP.441, HIS.348, ASP.346, ASP.440
**5**	− 10.4	SER.44, GLU.432, GLN.439, ASN.443	THR.517, ALA.349, GLN.531, ALA.454, ARG.437, ASP.441, GLN.438, PHE.516, ALA.514	ARG.450, GLN.349	HIS.348, ASP.440, ASP.346	PRO.528, LYS.352, HIS.515, ALA.451, ALA.444, LEU.433,
**6**	− 9.8	LYS.38, GLN.439, GLU.432	VAL.435, SER.44, HIS.515, ASN.443, TYR.41, TPR.409, ALA.349, THR.517, LEU.433, THR.445, ASP.441	ARG.450	ASP.440, ASP.346	LYS.352, HIS.348, ALA.451, ASP.40
**7**	− 9.2	HIS.348, ASN.443, TYR.41	ALA.451, ASN.447, THR.517, ALA.349, GLN439, GLN531, SER.436, VAL.435, GLU.37, SER.44, HIS.515, ALA.444, THR.445	ARG.450	ASP.440	ASP.441, LYS.352
**8**	− 10.0	GLN.439	ASN.447, LYS.38, GLU.37, TYR.41, ASP.40, GLN.531, PHE.516, HIS.515, ASN443, ASN.46, SER.44, ALA.349	LYS.352	ASP.440	ASP.346, HIS.348, ALA.354, ARG.450, ALA451, PRO.442, PRO.442, PRO.528, THR.517
**9**	− 10.5	LYS.352, ASN.46, GLN.439	ALA.349, ASP.40, THR.517, LYS.38, PRO.528, GLN.438, LEU.433, GLU.432, ALA.451, ARG.450, SER.44, ASN447	LYS352	ASP.440, ASP.441	ASP.346, ALA.444, ALA.454, ARG.437, HIS.348, PRO.442
**10**	− 10.9	LYS.352, THR.445	THR.448, PRO.442, ASN.447, ASP.440, VAL.435, GLU.37, ASP.40, SER.44, GLN.439, ASN.443, HIS.515, GLN.438, GLU.432	HIS.348, ARG.450	ASP.441, HIS.515	ALA.514, ALA.451, TYR.41, LYS.38, ALA.444, LEU.433, ALA454

In our docking study, compound 4 exhibited a strong binding affinity (-10.1 kcal/mol) with the target protein. Conventional hydrogen bonding involved GLU.432, ASN.443, and ALA.514, while diverse van der Waals interactions included ALA.454, ARG.437, LEU.433, GLN.438, THR.445, and HIS.515. Unfavorable positive-positive interactions with ARG.450 and GLN.439 were noted. Compound **4** displayed various π-interactions, such as π-cation/anion interactions with ASP.441, HIS.348, ASP.346, and ASP.440. Alkyl interactions with ALA.451 and π–alkyl interactions with ALA.444 and HIS.348 were observed, along with a unique π–π T-shaped interaction involving HIS.348 with the aromatic ring as shown in Fig. 8.

Similarly, compound **5** exhibits a − 10.4 kcal/mol, slightly higher than compound **4**. SER.44, GLN.349, ASN.443, and GLU.342 show conventional hydrogen bonding with the electronegative atoms (O, N, and S) of compound **5**. Compound **5** is also surrounded by van der Waals interactions, as shown in Table 4 and Fig. 8. ARG.450 and GLN.349 are involved in the unfavorable positive-positive interaction. HIS.348, ASP.440, and ASP.346 participate in attractive charges/π-cation interactions with the aromatic ring and NH species. ALA.444, LEU.433, and HIS.348 amino acids interact with the alkyl (–C_2_H_5_) group via π–alkyl interaction. Amino acids (LYS.352 and PRO.528) interact with the aromatic ring via π–interaction, while HIS.515 shows the π–stacked interaction.

Compound **6** exhibited a binding affinity of − 9.8 kcal/mol, involving conventional hydrogen bonds with LYS.38, GLN.439, and GLU.432. Van der Waals interactions included VAL.435, SER.44, HIS.515, ASN.443, TYR.41, and others. The ARG.450 shows the unfavorable positive-positive interaction nitrogen species. Attractive charges/π-cation interactions were observed with ASP.440 and ASP.346, while alkyl interactions involved ALA.444, HIS.348, and ALA.451.

In the case of compound **7**, it displayed a binding affinity of − 9.2 kcal/mol, forming conventional hydrogen bonds with HIS.348, ASN.443, and TYR.41, and engaging in van der Waals interactions with ALA.451, ASN.447, THR.517, and others. An unfavorable positive-positive interaction was noted with ARG.450. Attractive charges/π–cation interactions were observed with ASP.440, and alkyl interactions involved ASP.441, LYS.352, and HIS.515.

Compound **8** show the binding affinity of − 10.0 kcal/mol, establishing conventional hydrogen bonds with GLN.439 and participating in van der Waals interactions with ASN.447, LYS.38, GLU.37, TYR.41, and others. An unfavorable positive–positive interaction involved LYS.352. Attractive charges/π–cation interactions were observed with ASP.440, ASP.346, HIS.348, ALA.354, and others as shown in Table 4. THR.517 interacts with ring via π-sigma interaction as shown in Fig. 8.

Moving to compound **9**, it displayed a binding affinity of − 10.5 kcal/mol, involving conventional hydrogen bonds with LYS.352, ASN.46, and GLN.439, and participating in van der Waals interactions with ALA.349, ASP.40, THR.517, LYS.38, and others. An unfavorable positive-positive interaction was noted with LYS.352. Attractive charges/π–cation interactions were observed with ASP.440 and ASP.441, and alkyl interactions involved ASP.346, ALA.444, ALA.454, ARG.437, HIS.348, and PRO.442.

Finally, compound **10** exhibited the highest binding affinity of − 10.9 kcal/mol, forming conventional hydrogen bonds with LYS.352 and THR.445, and engaging in van der Waals interactions with THR.448, PRO.442, ASN.447, ASP.440, and others. An unfavorable positive-positive interaction was identified with HIS.348 and ARG.450. Attractive charges/π–cation interactions were observed with ASP.441 and HIS.515, while alkyl interactions involved ALA.514, ALA.451, TYR.41, LYS.38, ALA.444, LEU.433, and ALA.454.

In conclusion, our docking study has provided a comprehensive insight into the binding affinities and molecular interactions of compounds **4**–**10** with the target protein. Notable interactions, including conventional hydrogen bonding, van der Waals interactions, and attractive charges/π–cation interactions, contribute to the overall binding profiles. These findings contribute valuable information for further optimization and refinement of these compounds for enhanced binding efficacy and potential therapeutic applications.

## Methods

### General

All of the chemicals, reagents and solvents utilized in this study were of synthetic grade and purchased from BDH, Sigma-Aldrich, and Merck. Using the solvent system of *n*-hexane and ethyl acetate, thin layer chromatography (TLC) has been carried out on pre-coated silica gel 60 F254 aluminium cards. To assess the melting points of the synthesized compounds, Stuart SMP10 melting point apparatus was used. The masses of these analogues were determined using advanced high resolution electrospray ionization mass spectrometry (HR-ESI-MS) (Agilent 6530 LC Q-TOF, USA/EU, produced in Singapore). On a Bruker Avance 150 MHz and 600 MHz spectrophotometer (BRUKER, Zürich, Switzerland), the ^13^C-NMR and ^1^H-NMR spectra were captured using solvent peaks as internal references (DMSO-*d*_*6*_, δ_H_: 3.34; δ_C_: 39.9–39.1) respectively. The abbreviations s: singlet, d: doublet, t: triplet, m: multiplet, *J*: coupling constant (in Hz), and for chemical shifts in parts per million (ppm) are used in the explanation of NMR spectra.

### General procedure for the synthesis of compounds (1–10)

In the first step, 1,3-diethyl-2-thiobarbituric acid was refluxed with 2,4-dihydroxybenzaldehyde in a 100 ml round bottomed (RB) flask in the presence of catalytic amount of triethylamine (Et_3_N) in 100% ethanol for 2 h to obtain compound **1**, which was further refluxed for 3–4 h in DMF solvent with ethyl chloroacetate having catalytic amount of potassium carbonate (K_2_CO_3_) to get the desired compound **2**. Then, hydrazine hydrate was refluxed with compound **2** in ethanol to get the desired *bis*-hydrazide **3**. At last, different substituted aromatic aldehydes were refluxed for 4–5 h with compound **3** in ethanol solvent having 4–5 drops of acetic acid to get the desired *bis*-Schiff bases of thiobarbituric acid nucleus. Thin layer chromatographic technique was established to check the formation of product compounds using 30% polar system of *n*-hexane and ethyl acetate (7:3). After formation of the products, colored precipitates were appeared by pouring the mixtures to cold distilled water; the precipitates were washed with distilled water after filtration and kept overnight for drying. These product compounds were recrystallized with absolute ethanol to get them in pure form. Structures of all the compounds were deduced through HR-ESI-MS, ^13^C-, and ^1^H-NMR spectroscopic techniques.

### Spectral data of the synthesized compounds (4–10)

#### 2-(4-((1,3-diethyl-4,6-dioxo-2-thioxotetrahydropyrimidin-5(2*H)*-ylidene)methyl)-3-(2–2-(4-hydroxybenzylidene)hydrazinyl)-2-oxoethoxy)phenoxy)-*N*’-(4-hydroxybenzylidene)aceto hydrazide (4)

White Amorphous Solid; Yield: 78%; ^1^H-NMR (600 MHz, DMSO-*d*_*6*_): *δ* 11.46 (1H, s, –CH=), 9.35 (1H, s, –CH=N–), 9.30 (1H, s, –CH=N–), 9.18 (1H, s, –NH), 8.94 (1H, s, –NH), 8.60 (1H, s, –OH), 8.55 (1H, s, –OH), 8.10–6.14 (11H, m, Ar–H), 4.58 (2H, s, –CH_2_), 4.43 (2H, s, –CH_2_). ^13^C-NMR (150 MHz, DMSO-*d*_*6*_): *δ* 166.5, 166.3, 166.2, 161.6, 158.5, 157.3, 155.5, 146.6, 135.1, 132.9, 130.9, 130.5, 129.6, 128.3, 127.5, 126.9, 126.4, 125.8, 124.3, 118.2, 115.6, 107.8, 107.7, 107.2, 100.3, 100.2, 67.0, 66.7, 66.6, 66.4, 39.9, 39.6, 39.2, 39.0. HRMS (ESI^+^): [M + Na]^+^ calcd for C_33_H_32_N_6_NaO_8_S: 697.0477; found 697.0482.

#### 2-(4-((1,3-diethyl-4,6-dioxo-2-thioxotetrahydropyrimidin-5(2*H)*-ylidene)methyl)-3-(2–2-(2-nitrobenzylidene)hydrazinyl)-2-oxoethoxy)phenoxy)-*N*’-(2-nitrobenzylidene)aceto hydrazide (5)

Off-White Amorphous Solid; Yield: 80%; ^1^H-NMR (600 MHz, DMSO-*d*_*6*_); *δ* 10.08 (2H, s, 2 × –NH), 9.90 (1H, s, –CH=), 9.20 (2H, s, 2 × –CH=N–), 8.31–6.50 (11H, m, Ar–H), 5.19–4.35 (4H, m, –CH_2_). ^13^C-NMR (150 MHz, DMSO-*d*_*6*_): *δ* 168.3, 166.5, 166.2, 161.4, 160.7, 160.4, 160.3, 159.3, 158.4, 158.2, 157.2, 155.5, 139.5, 135.1, 130.2, 129.6, 129.0, 128.7, 127.4, 126.7, 125.9, 125.1, 125.0, 118.2, 115.8, 115.7, 115.3, 107.7, 107.2, 100.3, 100.2, 67.0, 66.4, 39.8, 39.4, 39.1, 39.0. HRMS (ESI^+^): [M + K]^+^ calcd for C_33_H_30_N_8_KO_10_S: 769.2839 found 769.2817.

#### 2-(4-((1,3-diethyl-4,6-dioxo-2-thioxotetrahydropyrimidin-5(2*H)*-ylidene)methyl)-3-(2–2-(4-nitrobenzylidene)hydrazinyl)-2-oxoethoxy)phenoxy)-*N*’-(4-nitrobenzylidene)aceto hydrazide (6)

White Amorphous Solid; Yield: 90%; ^1^H-NMR (600 MHz, DMSO-*d*_*6*_); *δ* 11.47 (1H, s, –CH=), 11.38 (1H, s, –CH=N–), 11.36 (1H, s, –CH=N–), 9.57 (1H, s, –NH), 9.55 (1H, s, –NH), 8.30–6.52 (11H, m, Ar–H), 4.60 (2H, s, –CH_2_), 4.35 (2H, s, -CH_2_). ^13^C-NMR (150 MHz, DMSO-*d*_*6*_): *δ* 166.3, 166.2, 162.2, 160.2, 159.6, 158.8, 149.0, 148.6, 145.6, 143.5, 140.1, 139.4, 134.2, 129.6, 129.2, 125.3, 124.1, 124.0, 115.1, 107.9, 100.2, 67.0, 66.4, 39.9, 39.4, 39.2, 39.0. HRMS (ESI^+^): [M + H]^+^ calcd for C_33_H_34_N_9_O_10_S: 748.2439; found 748.2477.

#### 2-(4-((1,3-diethyl-4,6-dioxo-2-thioxotetrahydropyrimidin-5(2*H)*-ylidene)methyl)-3-(2–2-(3-nitrobenzylidene)hydrazinyl)-2-oxoethoxy)phenoxy)-*N*’-(3-nitrobenzylidene)aceto hydrazide (7)

White Amorphous Solid; Yield: 84%; ^1^H-NMR (600 MHz, DMSO-*d*_*6*_); *δ* 11.79 (1H, s, –CH=), 11.54 (2H, s, –CH=N–), 9.57 (1H, s, –NH), 9.32 (1H, s, –NH), 8.39–6.60 (11H, m, Ar–H), 5.17 (4H, s, –CH_2_). ^13^C-NMR (150 MHz, DMSO-*d*_*6*_): *δ* 166.3, 162.1, 160.5, 159.4, 158.6, 157.4, 148.2, 138.5, 135.7, 135.2, 134.9, 134.4, 134.2, 131.1, 130.6, 130.0, 129.6, 126.9, 125.8, 125.3, 122.6, 122.2, 121.4, 118.8, 107.9, 100.2, 67.0, 66.4, 64.9, 39.8, 39.6, 39.3, 39.0. HRMS (ESI^+^): [M + NH_4_]^+^ calcd for C_33_H_34_N_9_O_10_S: 748.2539; found 748.2543.

#### *N*’-(2,4-dichlorobenzylidene)-2-(5-(2-(2-(2,4-dichlorobenzylidene)hydrazinyl)-2-oxoethoxy)-2-((1,3-diethyl-4,6-dioxo-2-thioxotetrahydropyrimidin-5(2*H)*-ylidene)methyl)phenoxy) acetohydrazide (8)

Pale yellow Amorphous Solid; Yield: 86%; ^1^H-NMR (600 MHz, DMSO-*d*_*6*_); *δ*

11.43 (1H, s, –CH=), 9.54 (1H, s, –NH), 9.35 (1H, s, –NH), 9.30 (1H, s, –CH=N–), 9.28 (1H, s, –CH=N–), 8.32–6.50 (11H, m, Ar–H), 4.59 (2H, s, –CH_2_), 4.47 (2H, s, –CH_2_). ^13^C-NMR (150 MHz, DMSO-*d*_*6*_): *δ* 166.5, 166.4, 161.6, 158.2, 157.3, 129.5, 115.6, 107.8, 100.3, 67.0, 66.4, 39.9, 39.7, 39.4, 39.1. HRMS (ESI^+^): [M + NH_4_]^+^ calcd for C_33_H_32_Cl_4_N_7_O_6_S: 793.2595; found 793.2653.

#### *N*’-(2,3-dichlorobenzylidene)-2-(5-(2-(2-(2,3-dichlorobenzylidene)hydrazinyl)-2-oxoethoxy)-2-((1,3-diethyl-4,6-dioxo-2-thioxotetrahydropyrimidin-5(2*H)*-ylidene)methyl)phenoxy) acetohydrazide (9)

Yellow Amorphous Solid; Yield: 82%; ^1^H-NMR (600 MHz, DMSO-*d*_*6*_); *δ* 11.89 (1H, s, –CH=), 9.52 (1H, s, –NH), 9.33 (1H, s, –NH), 8.93 (2H, s, –CH=N–), 8.03 (2H, d, *J* = 7.2 Hz, Ar–H), 7.86 (2H, d, *J* = 8.4 Hz, Ar–H), 7.73 (2H, d, *J* = 7.8 Hz, Ar–H), 7.42 (1H, t, *J* = 7.8 Hz, Ar–H), 4.57–4.32 (4H, m, –CH_2_). ^13^C-NMR (150 MHz, DMSO-*d*_*6*_): *δ* 166.3, 166.1, 162.2, 159.8, 158.8, 156.4, 133.5, 132.6, 132.1, 129.7, 128.6, 127.9, 126.9, 123.8, 115.0, 107.9, 100.2, 66.9, 66.4, 39.9, 39.5, 39.1. HRMS (ESI^+^): [M + NH_4_]^+^ calcd for C_33_H_32_Cl_4_N_7_O_6_S: 793.2599; found 793.2661.

#### *N*’-(3-chlorobenzylidene)-2-(5-(2-(2-(3-chlorobenzylidene)hydrazinyl)-2-oxoethoxy)-2-((1,3-diethyl-4,6-dioxo-2-thioxotetrahydropyrimidin-5(2*H)*-ylidene)methyl)phenoxy)aceto hydrazide (10)

Ash-White Amorphous Solid; Yield: 80%; ^1^H-NMR (600 MHz, DMSO-*d*_*6*_); *δ* 11.74 (1H, s, –CH=), 9.50 (1H, s, –NH), 9.27 (1H, s, –NH), 8.89 (1H, s, –CH=N–), 8.83 (1H, s, –CH=N), 8.38–6.57 (11H, m, Ar–H), 5.10 (2H, s, –CH_2_), 4.52–4.28 (2H, m, –CH_2_). ^13^C-NMR (150 MHz, DMSO-*d*_*6*_): *δ* 168.1, 166.2, 162.0, 160.4, 159.3, 158.6, 157.3, 148.1, 138.5, 135.7, 134.7, 134.4, 131.0, 130.6, 129.9, 129.5, 125.7, 122.6, 121.3, 118.7, 115.1, 107.8, 100.1, 66.9, 39.8, 39.4, 39.0. HRMS (ESI^+^): [M + K]^+^ calcd for C_33_H_30_Cl_2_KN_6_O_6_: 748.2495; found 748.2488.

### Computational details

All the density functional theory (DFT) calculations were carried out at Gaussian 09 package. The geometry optimizations were performed using a hybrid functional B3LYP with 6-311++G(d,p) basis set^42,43^. To further ensure the true minima, the frequency calculations were performed without imaginary frequency. For the account of intramolecular interaction the DFT-D3 approach was utilized. The NMR calculations were performed by Gauge-Invariant Atomic Orbital (GIAO) method at B3LYP/6-311+G(2d,p)^44,45^. The detailed electronic structural analysis was performed at TD-DFT using CAM-B3LYP/6-311++G(d,p) method. For the prediction of electrophilic and nucleophilic sites of synthesized compounds the molecular electrostatic potential (MEP) was performed. Furthermore, docking analysis was done by using AutoDock Vina^46^ to investigates the binding between amino acids residues and protein (α-glucosidase). For docking analysis, the dimension of grid box was selected as 40, 40, and 40 in *x, y,* and *z-*direction with grid-point spacing of 0.375 Å.

## Conclusions

In conclusions, we have reported impressive novel α-glucosidase inhibitors through the systematic synthesis of *bis*-Schiff base derivatives based on thiobarbituric acid scaffold. These derivatives were structurally confirmed through different spectroscopic techniques including HR-ESI-MS, ^1^H-, and ^13^C-NMR and finally screened them for their in vitro α-glucosidase inhibitory activity. In the series, compounds **8** and **9** displayed superior efficacy, better than the reference drug acarbose. These results highlight the possibility of thiobarbituric acid derivatives as effective candidates for the development of anti-diabetic medications. To move these derivatives closer to clinical use and support current attempts to find safe and effective diabetes therapies more research into their pharmacokinetics and safety profiles is necessary. Additionally, we have performed the docking study to elucidate the molecular interactions between these compounds and the target protein, offering a deeper understanding of their inhibitory mechanisms.

### Supplementary Information

Supplementary Information.

## Data Availability

All data generated or analyzed during this study are included in this published article and Supplementary Materials.
