# Supplementary material for: Synthesis, molecular docking and DFT analysis of novel bis-Schiff base derivatives with thiobarbituric acid for α-glucosidase inhibition assessment
Source: Sci Rep. 2024 Feb 10;14:3419. doi: 10.1038/s41598-024-54021-z (PMC10858901; doi:10.1038/s41598-024-54021-z)
Supplement: Supplementary file 1 — Supplementary Information. [file 41598_2024_54021_MOESM1_ESM.docx]

**Synthesis, Molecular Docking and DFT Analysis of Novel Bis-Schiff Base Derivatives with Thiobarbituric Acid for α-Glucosidase Inhibition Assessment**

Saba Gul^a1^, Faheem Jan^b,c1^, Aftab Alam^d^, Abdul Shakoor^a^, Ajmal Khan^e^, Abdullah F. AlAsmari^f^, Fawaz Alasmari^f^, Momin Khan^a^*, Li Bo^g^*

^a^Department of Chemistry, Abdul Wali Khan University, Mardan-23200, Pakistan

^b^Shenyang national laboratory for Materials Science, Institute of Metal Research Chineses Academy of Sciences, Shenyang, 110016, Liaoning, China

^c^School of Materials Science and Engineering, University of Science and Technology of China, Shenyang 110016, Liaoning, China

^d^Department of Chemistry, University of Malakand, P.O. Box 18800, Dir Lower, Pakistan

^e^Natural and Medical Sciences Research Center, University of Nizwa, PO Box 33, 616 Birkat Al Mauz, Nizwa, Oman

^f^Department of Pharmacology and Toxicology, College of Pharmacy, King Saud University, Riyadh 11451, Saudi Arabia

^g^Institute of Catalysis for Energy and Environment, College of Chemistry and Chemical Engineering, Shenyang Normal University, Shenyang 110034, China

**Corresponding Author:** [mominkhan@awkum.edu.pk](mailto:mominkhan@awkum.edu.pk); [boli@synu.edu.cn](mailto:boli@synu.edu.cn)

^1^Saba Gul and Faheem Jan equally contributed to this work.

**Supporting Information**

**
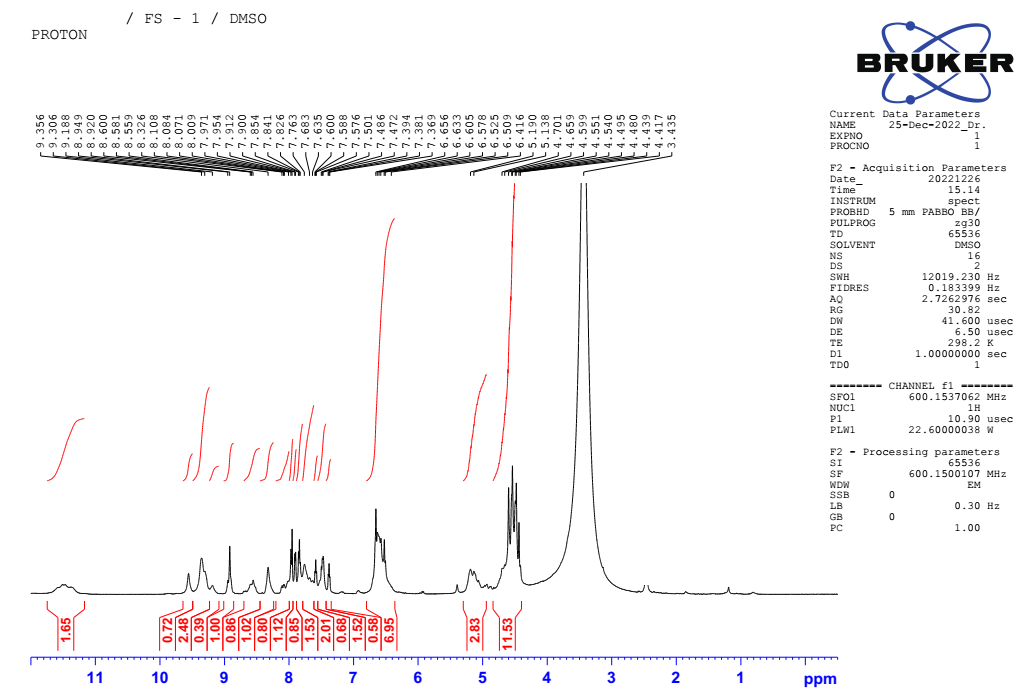
**

**
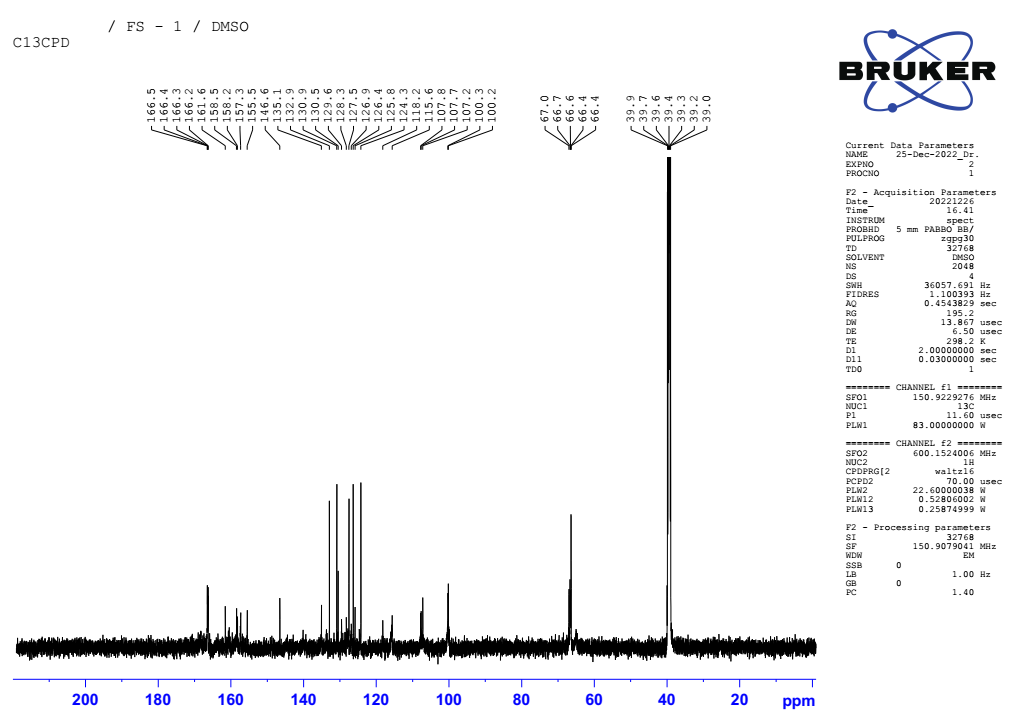
**


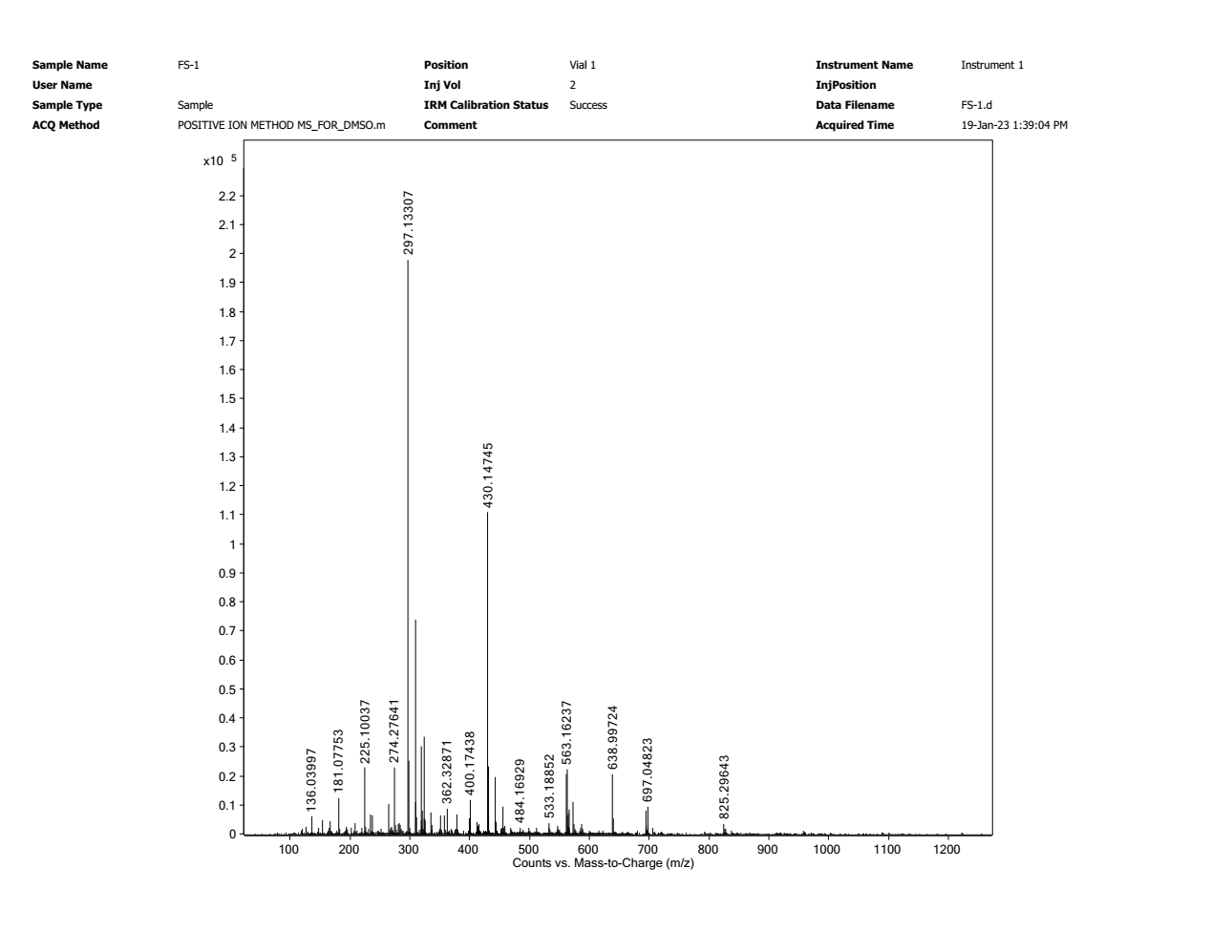


**Figure-1:** ^1^H-, ^13^C-NMR and HR-ESI-MS spectra of compound 4


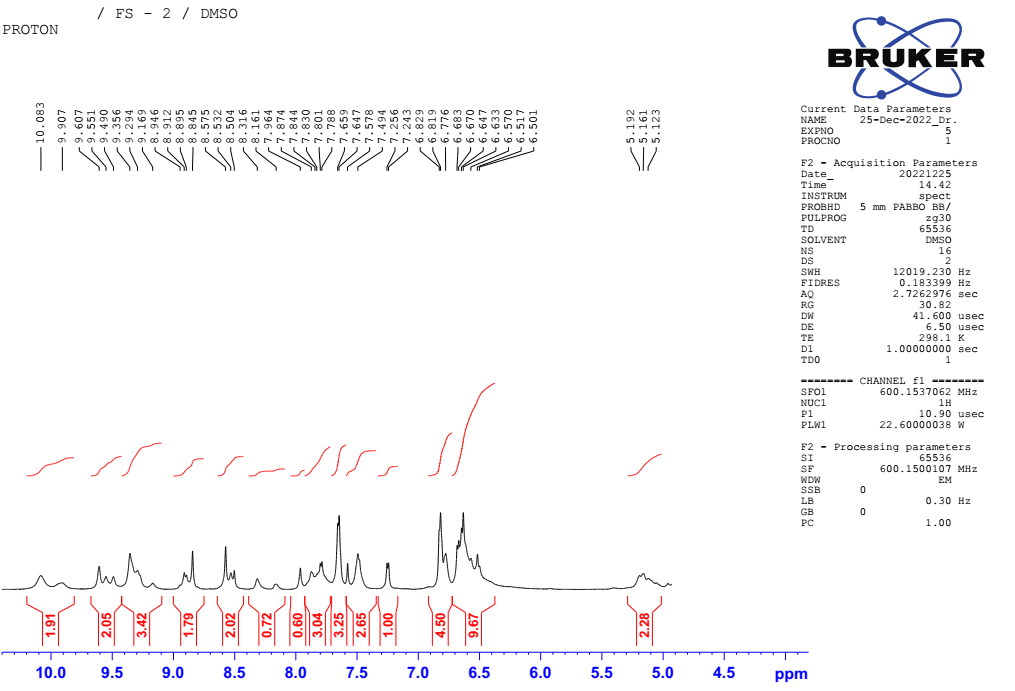


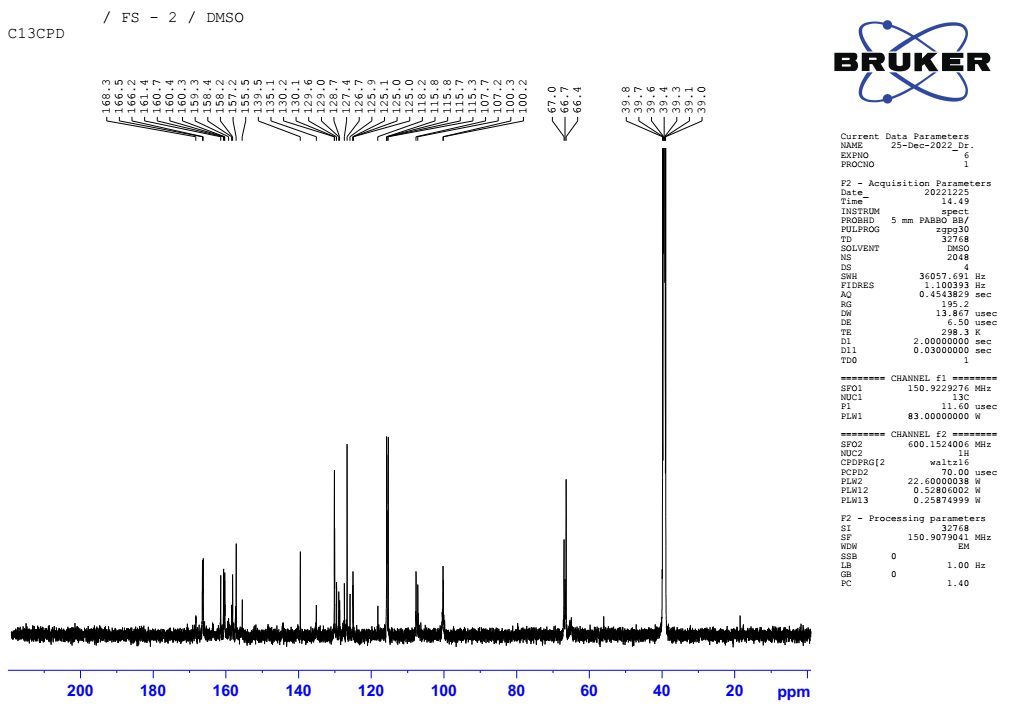


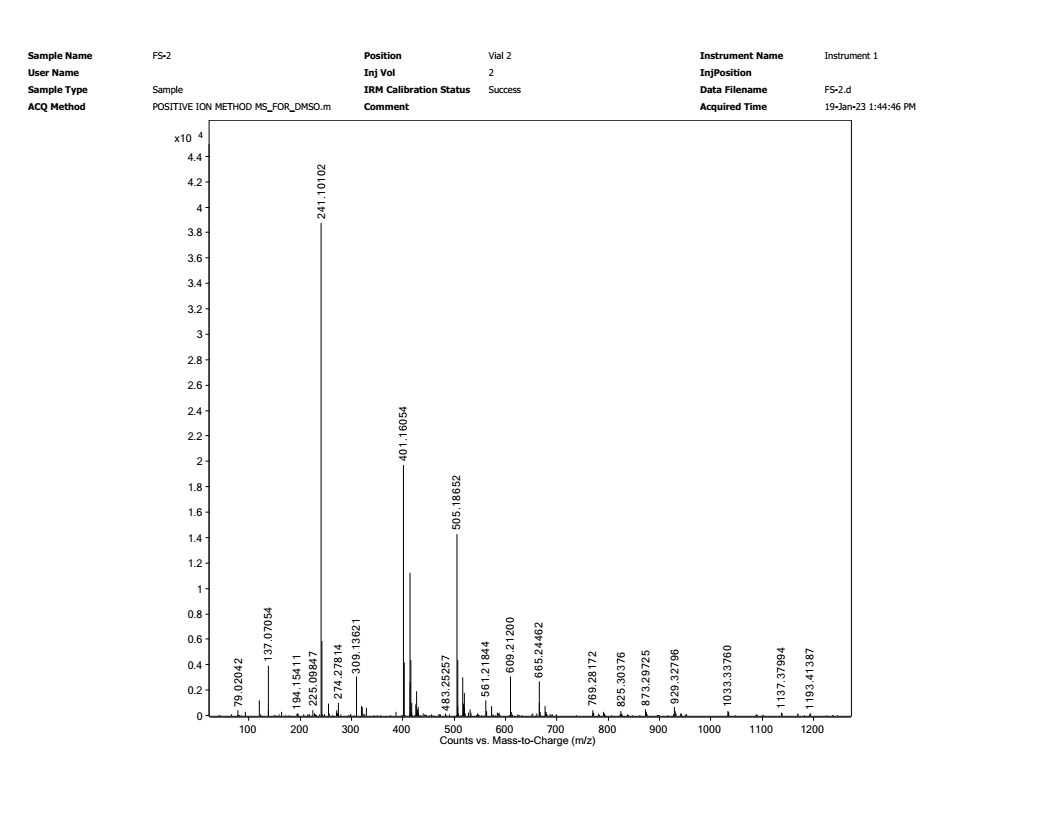


**Figure-2:** ^1^H-, ^13^C-NMR and HR-ESI-MS spectra of compound 5


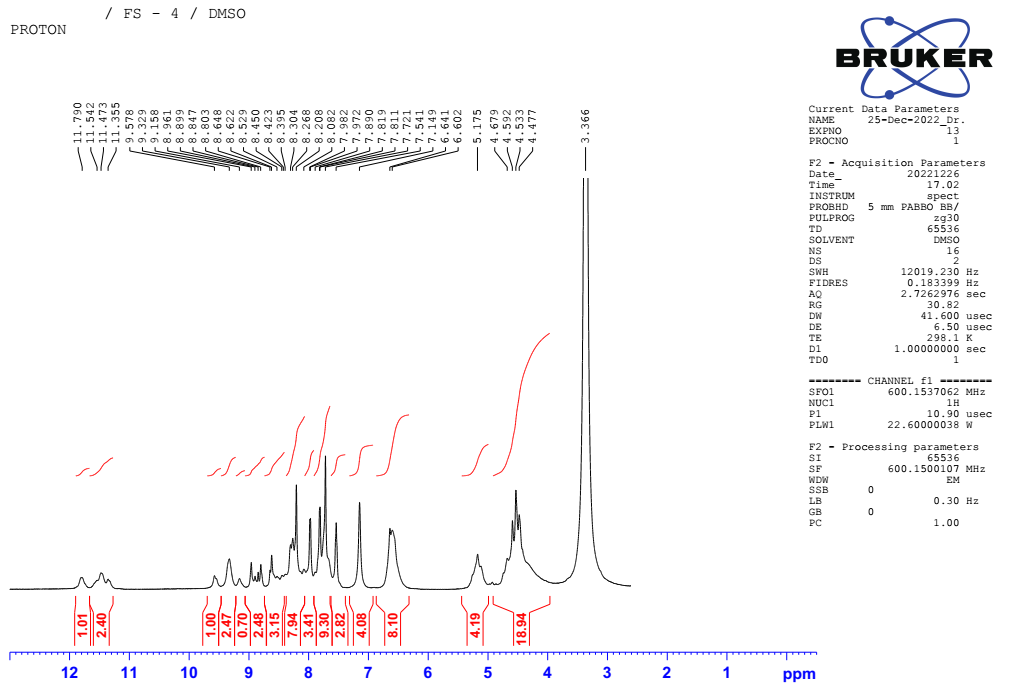


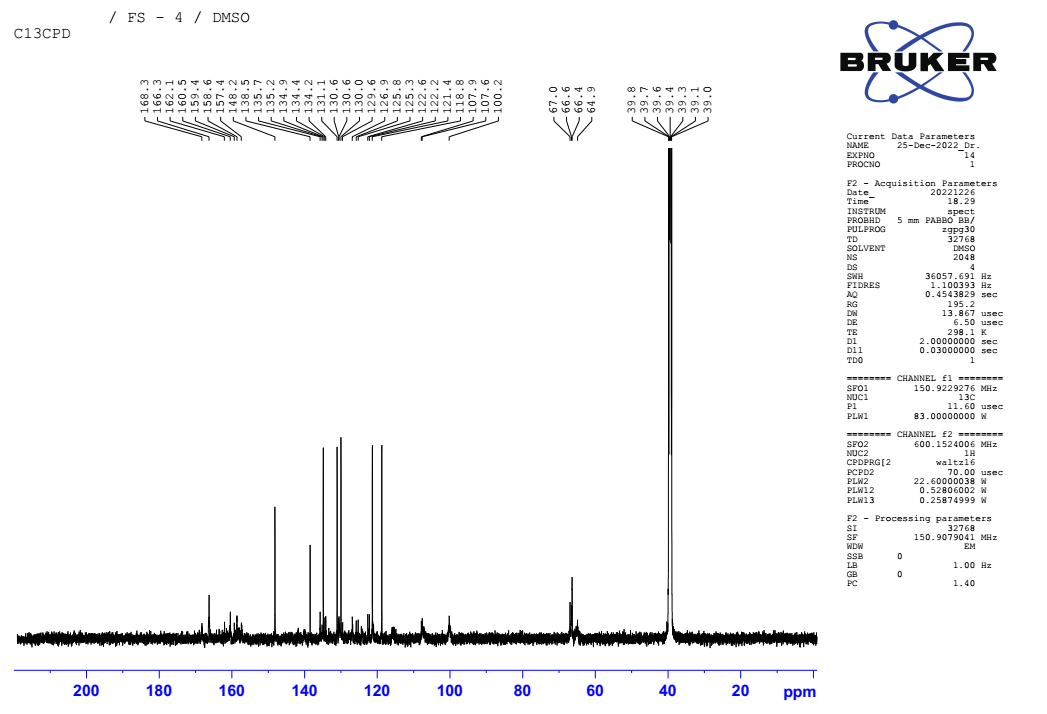


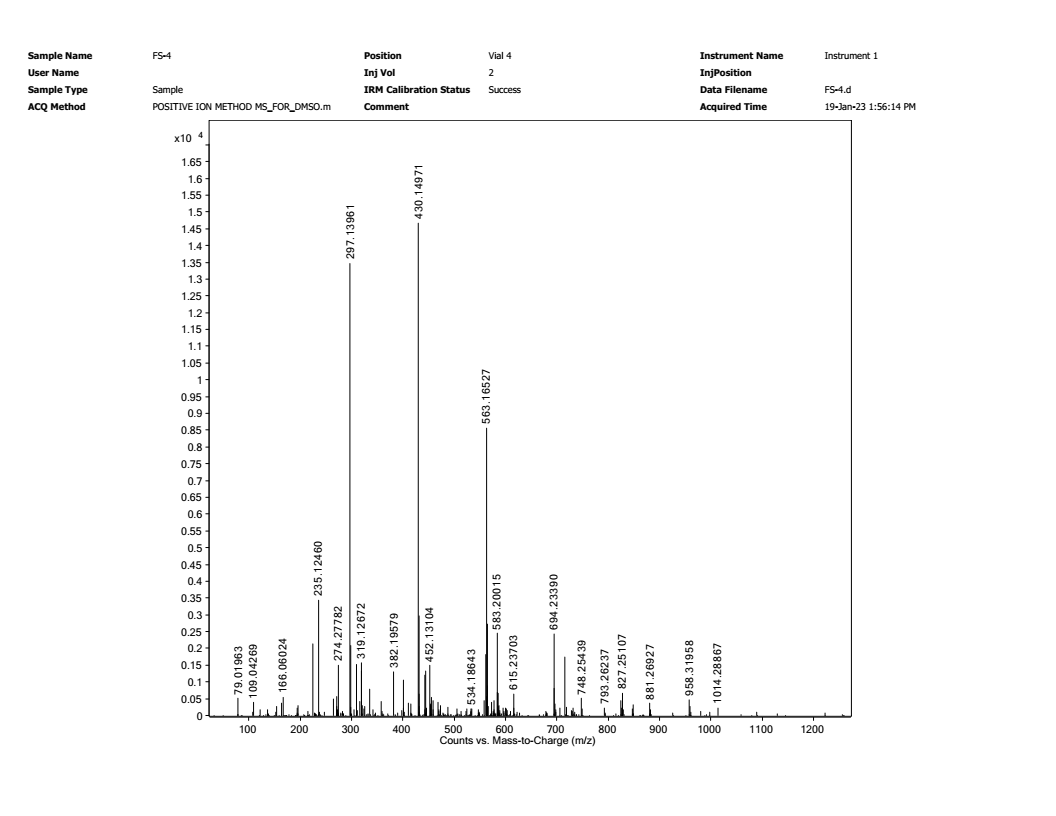


**Figure-3:** ^1^H-, ^13^C-NMR and HR-ESI-MS spectra of compound 7


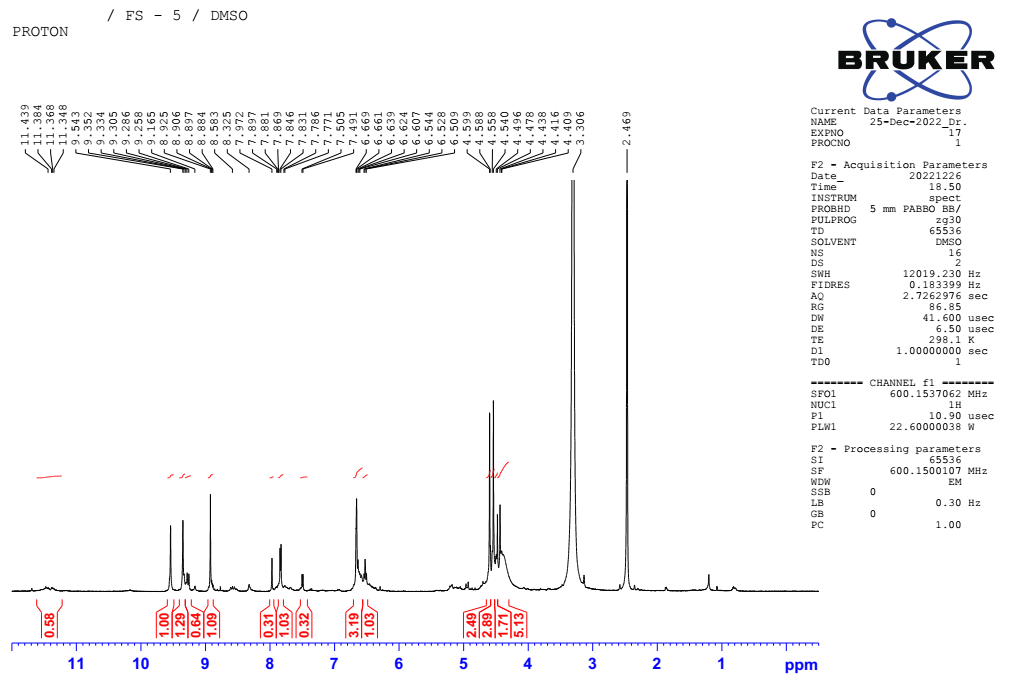


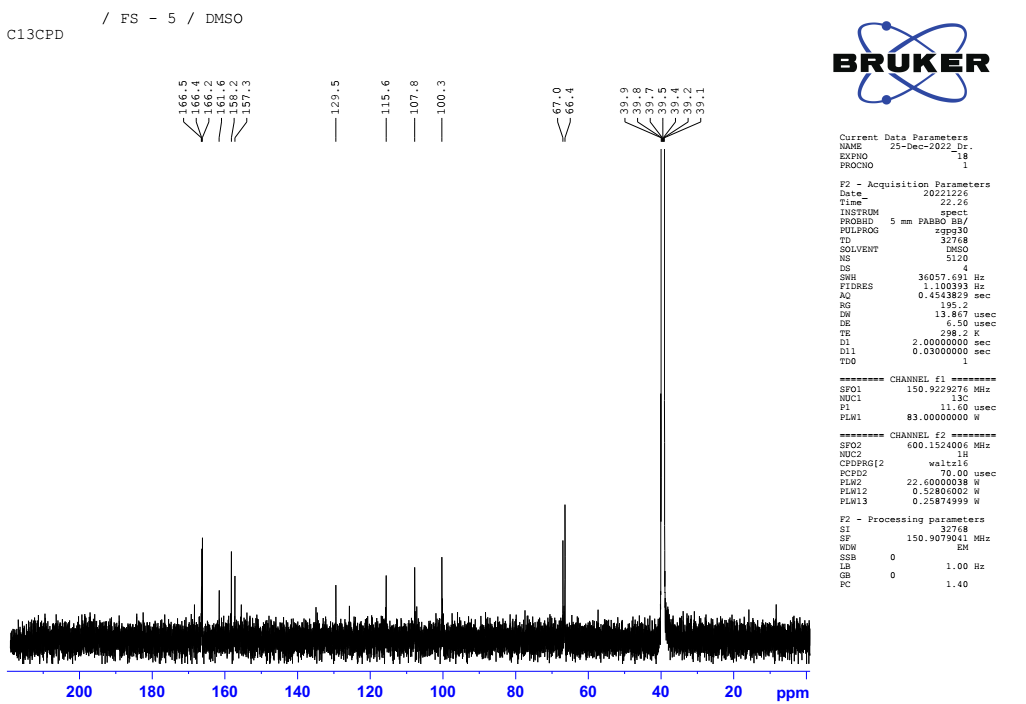


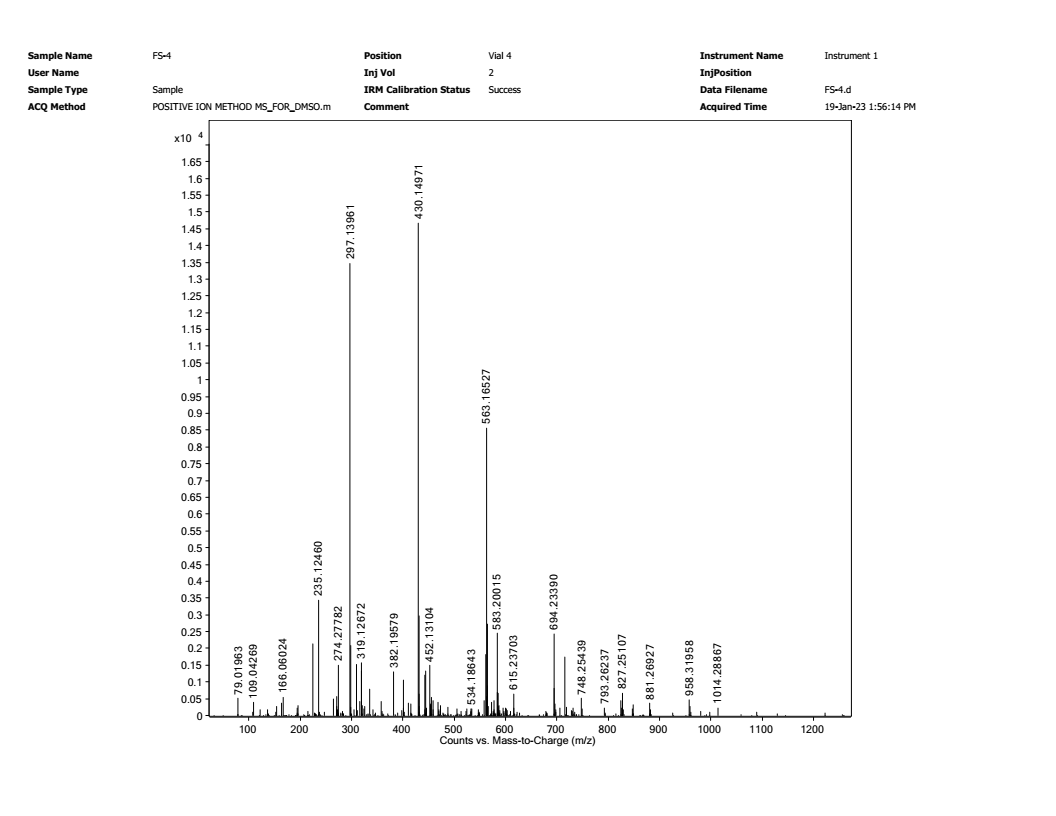


**Figure-4:** ^1^H-, ^13^C-NMR and HR-ESI-MS spectra of compound 8


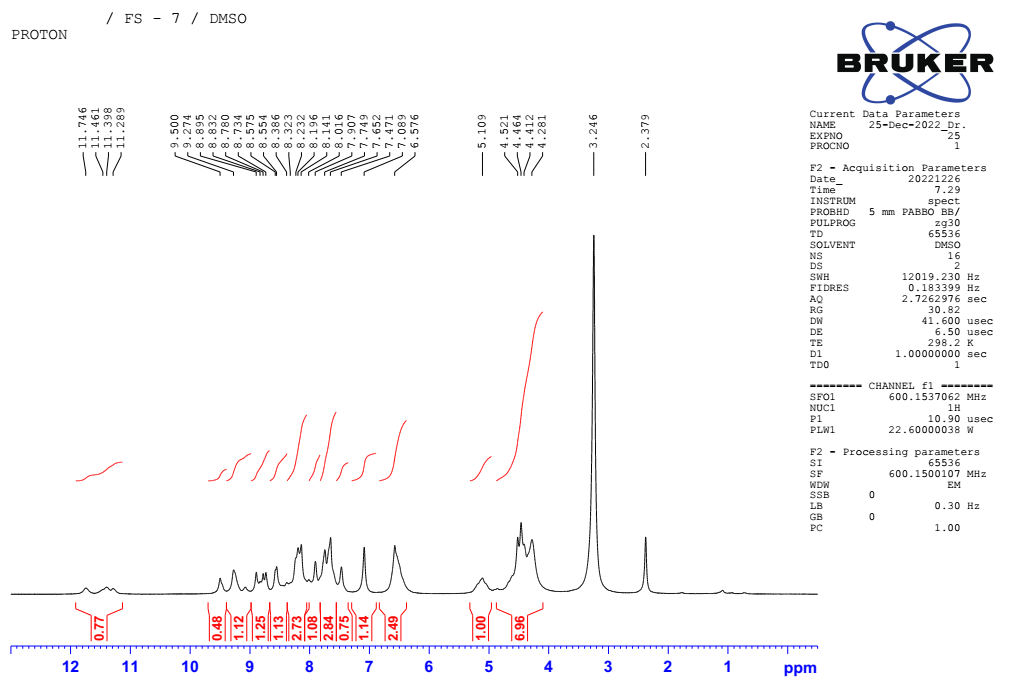


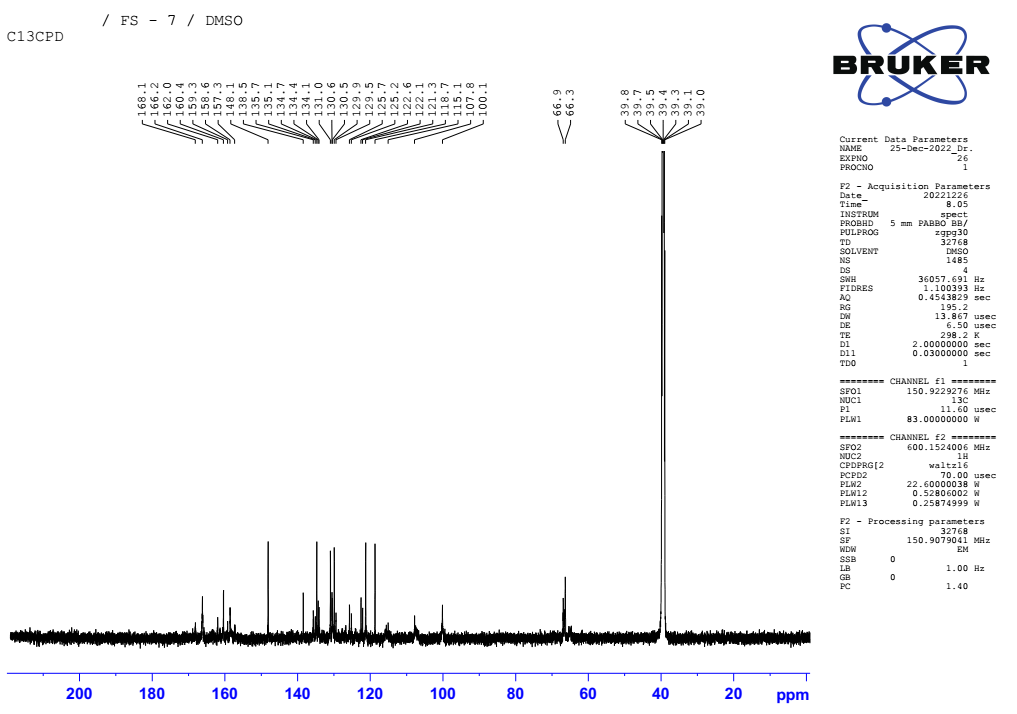


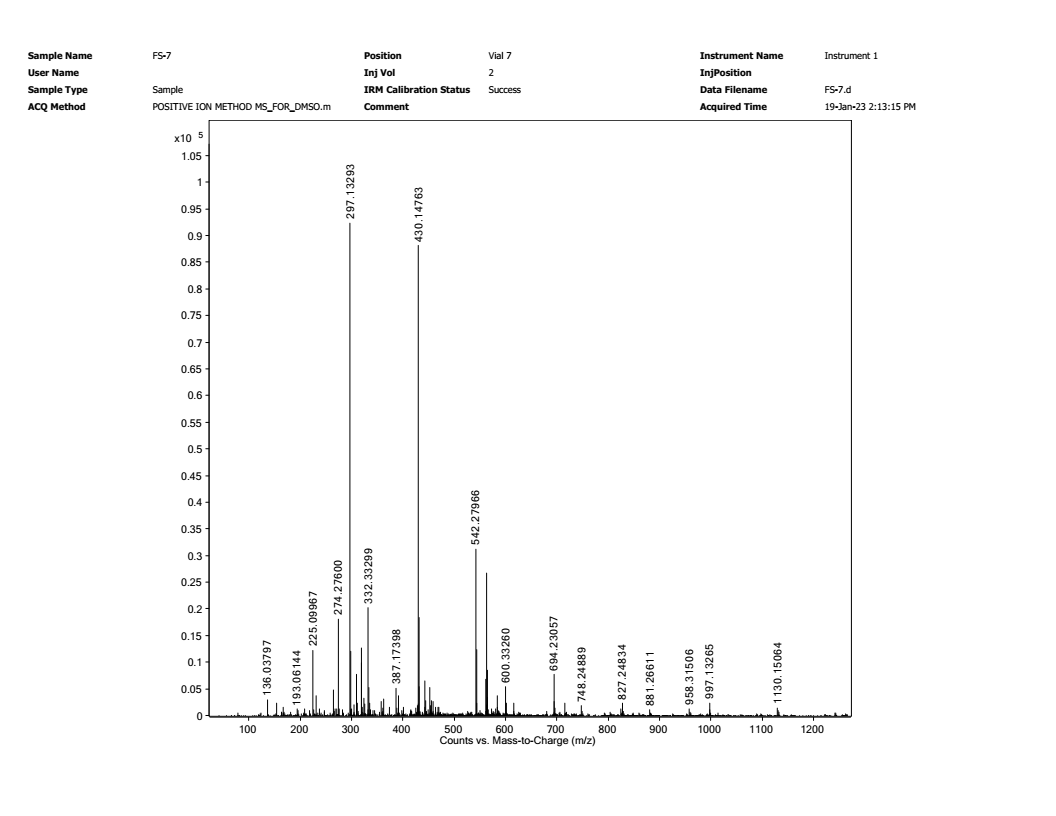


**Figure-5:** ^1^H-, ^13^C-NMR and HR-ESI-MS spectra of compound 10
